# Late‐onset Focal Dermal Elastosis—dermatoscopic and ultrasonographic assessment of this rare entity and literature review

**DOI:** 10.1111/srt.13461

**Published:** 2023-09-21

**Authors:** Katarzyna Korecka, Joanna Czuwara, Marta Szymoniak‐Lipska, Adriana Polańska, Ryszard Żaba, Aleksandra Dańczak‐Pazdrowska

**Affiliations:** ^1^ Department of Dermatology and Venereology Poznań University of Medical Sciences Poznań Poland; ^2^ Department of Dermatology Medical University of Warsaw Warsaw Poland; ^3^ Department of Dermatology Poznań University of Medical Sciences Poznań Poland

Dear Editor,

Late Onset Focal Dermal Elastosis (LOFDE) is a rare, acquired cutaneous entity. Clinically, it usually presents as multiple, small, flat, white to pale yellow papules located on the sides of the neck and flexural areas.^1^, ^2^ The lesions might be asymptomatic or pruritic and generally occur in the elderly skin. Mainly women are affected.^3^ In several reports regarding LOFDE, only a single case was described in a male patient.^4^ The histopathological hallmark of LOFDE is the accumulation of the structurally normal elastic tissue in the mid and deep reticular dermis forming conglomerates pushing up collagen bundles.^3^ Although LOFDE might clinically resemble pseudoxanthoma elasticum (PXE), their histopathological features differ, and no systemic disorders have been found in this condition. Other differential diagnoses include elastofibroma, papillary dermal elastolysis, nevus elasticus and Buschke‐Ollendorff syndrome.^1^ The pathogenesis of this entity is not known; however, some theories have been proposed. Since there are no clues of solar elastosis in histopathology, ageing not related to sun damage might have an impact on the appearance of the skin lesions.^3^ The loss of an age‐related growth‐regulating gene control mechanism leading to the overproduction of structurally healthy tissue has been described in this entity.^5^ On the other hand, overexpression of elastin has also been proposed.^4^ The changes in this entity are irreversible and the treatment is targeted to relief sometimes concomitant pruritus.^3^

Our patient is a 57‐year‐old woman presenting with multiple, asymptomatic, unilateral yellowish papules located predominantly on the skin of the right lower extremity and groin. Single lesions were noticed on the right part of the abdomen (Figure 1A,B). The accentuation of the papules was marked after skin stretching. Furthermore, the lesions did not cross the middle line of the body. They appeared about 3–4 years prior to the visit, although the patient was not sure of the duration since she noticed them once they had been spread.

**FIGURE 1:**
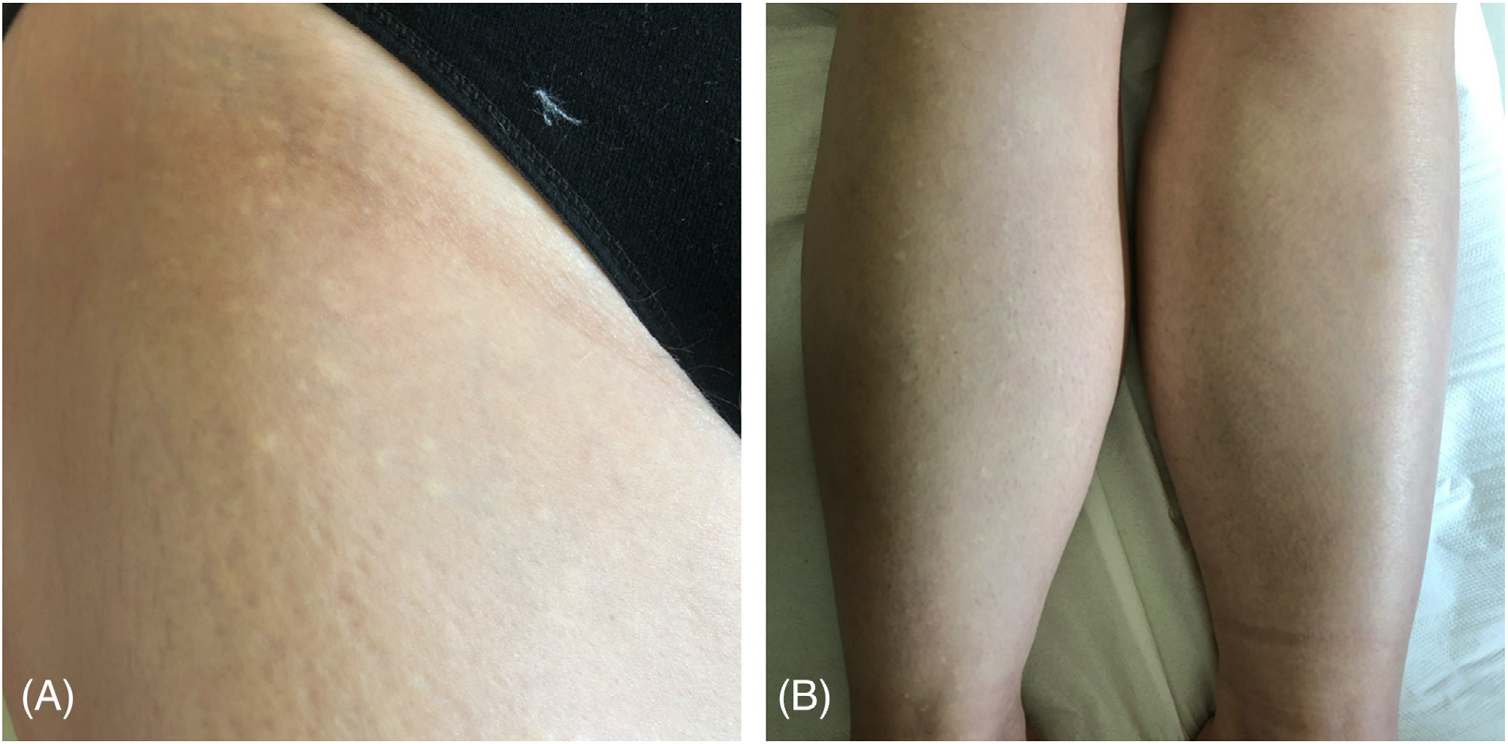
Clinical image of LOFDE: (A) multiple white papules on the right thigh, (B) multiple white papules located unilaterally on the right shin.

On dermatoscopy (DermLite DL5, 10x magnification) white globules, corresponding to the accumulation of the connective tissue, presumably elastic tissue pushing collagen bundles were noticeable (Figure 2A). They were also visible as bright, blue globules in the Ultraviolet‐ Induced Fluorescence Dermatoscopy (Figure 2B). In high frequency ultrasound (HFUS, DermaScan Cortex C, 20 MHz) hypoechogenic round‐shaped lesion with poorly demarcated borders covered by hyperechogenic entrance echo were observed (Figure 2C). A 4 mm punch skin biopsy was taken, and histopathology revealed normal epidermis covered with lamellar keratin. The mid and deep reticular dermis was dense with thickened fibrillar bundles corresponding with the papule (Figure 3A). In orcein staining pink bundles were detected as structurally normal, increased in amount, dispersed between collagen bundles elastic fibers (Figure 3B). No fragmentation, phagocytosis, histiocytic or inflammatory response was noticeable. The pathological findings were consistent with focal dermal elastosis, and in correlation with clinical presentation in the 57‐yo woman as late‐onset FDE.

**FIGURE 2:**
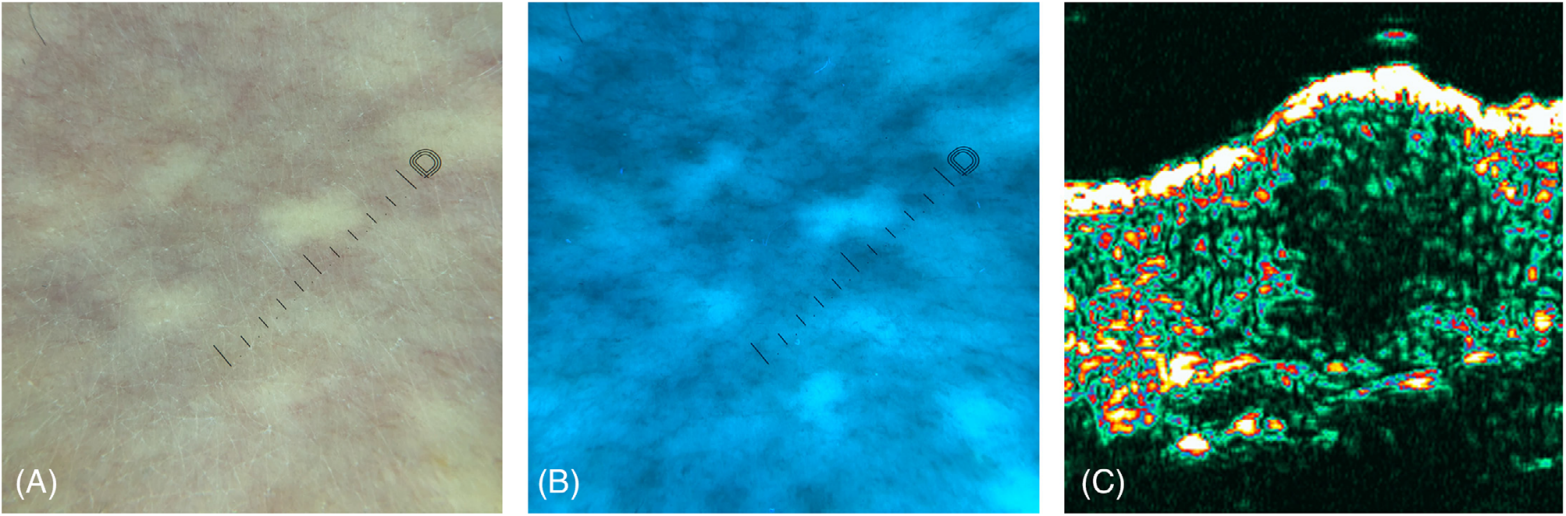
(A) Dermatoscopy of LOFDE lesions (DermLite DL5, magnification 10×): white round structureless areas on an erythematous background, (B) bright, blue, irregular, sharply demarcated patches in the Ultraviolet‐ Induced Fluorescence Dermatoscopy (DermLite DL5, magnification 10x), (C) hypoechogenic, round‐shaped lesion with poorly demarcated borders covered by hyperechogenic entrance echo seen in HFUS (DermaScan Cortex C, 20 MHz).

**FIGURE 3:**
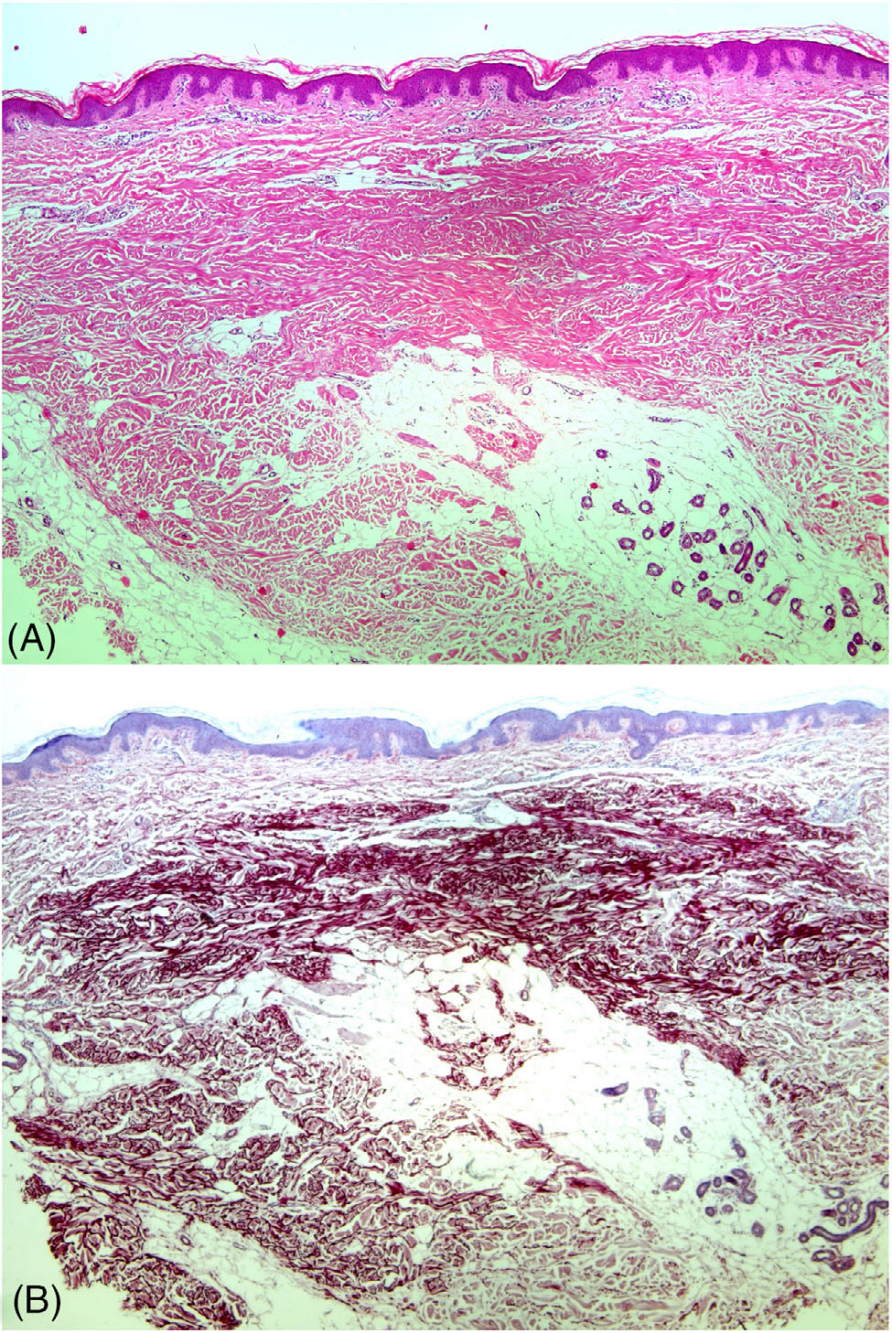
(A) H&E image of the lesion biopsy presents preserved epidermis and reticular dermis with densely packed collagen bundles, 100x; (B) orcein staining highlights the focal increase of structurally normal elastic fibers dispersed between collagen bundles, 100x.

To our knowledge, this is one of the very few described cases of late‐onset focal dermal elastosis, and the first one analyzed with non‐invasive skin imaging techniques such as dermatoscopy and high‐frequency ultrasound. A review of the available LOFDE cases in the literature is presented in Table 1.

**TABLE 1:** Cases of late‐onset focal dermal elastosis available from the literature.

Author/Ref	Year	Patient's age	Sex	Clinical presentation	Non‐invasive skin imaging
Tajima et al.	1995	85	Male	Multiple yellow papules on thighs, groins, popliteal fossae and antecubital fossae.	No
Tajima et al.	1995	65	Female	Asymptomatic papular lesions on the neck, thighs, antecubital fossae, groins and popliteal fossae.	No
Tajima et al.	1996	18	Female	Asymptomatic multiple yellow papules on the neck, inguinal area and axilla.	No
Limas et al.	1999	72	Female	Multiple yellowish papules on sides of the neck, axillae and antecubital fossae.	No
Limas et al.	1999	69	Female	Multiple yellowish papules on the sides of the neck.	No
Kossard et al.	2005	73	Female	Flat yellow lesions forming a cobblestone pattern over the neck, upper trunk and axillae.	No
Higgins et al.	2010	87	Female	Yellow, pruritic plaque on the posterior neck.	No
Tian et al.	2012	72	Female	Multiple, pale‐yellow papules on both sides of the neck, as well as the anterior part of the chest.	No
Camacho et al.	2012	48	Female	Multiple yellowish, symmetrically distributed papules on the dorsum of the hands.	No
Camacho et al.	2012	53	Female	Multiple yellowish, symmetrically distributed papules on the dorsum of the hands.	No
Wang et al.	2012	75	Female	Symmetrically distributed, white‐to‐yellow, non‐follicular papules on the posterolateral neck, anterior chest and axillae.	No
Wang et al.	2012	39	Female	Asymptomatic flesh‐colored lesions on the posterior neck, back, antecubital and popliteal fossae, thighs, forearms and wrists.	No
Chappell et al.	2016	54	Female	Firm, yellow, dermal papules located on the neck, antecubital and popliteal fossae, flexor surface of both forearms, and inner aspect of the thighs.	No
Hanami et al.	2018	73	Female	Whitish yellow papules and coalesced plaques on the axilla, trunk and extremities.	No
Knapp et al.	2020	59	Female	Flat, yellowish subcutaneous nodules, bilaterally over the patient's anterior shoulders, antecubital fossae, and thighs.	No
Fantini et al.	2022	70	Female	Yellow‐white papules symmetrically distributed along the posterior axillary folds.	No
Our case	2023	57	Female	Unilateral multiple, asymptomatic yellowish papules located predominantly on the skin of the right lower extremity, groin and right side of the abdomen.	Yes

Considering the fact that the pathogenesis of LOFDE has not been determined, it might be an underreported entity due to its peculiar clinical presentation. LOFDE papules clinically strongly mimic pseudoxanthoma elasticum (PXE) as well as the fibrotic phase of guttate morphea. In dermatoscopy, PXE reveals yellow‐to‐white clods on a reddish or whitish background, along with linear, irregular vessels,^6^, ^7^ whereas in HFUS, oval, homogeneous, hypoechogenic areas in the mid and deep dermis, undulating skin surface with regular epidermis, and normoechogenic, interpapular dermis can be seen. According to Guérin‐Moreau et al., PXE skin is primarily hypoechogenic.^8^ In the case of LOFDE, dermatoscopy presents white globules (better attenuated in the Ultraviolet‐Induced Reflectance Dermatoscopy) on an erythemateous background, while HFUS shows the separation of the collagen fibers in the reticular dermis as hypoechoic areas (corresponding to the accumulation of the elastic fibers as histology confirmed), similarly as HFUS has shown low‐reflection echoes in the deep dermis due to fat lobules herniations into atrophic skin, in for example, dermal striae.^9^ Histology with elastic tissue stain will emphasize the distinctive elastic fibers arrangement in LOFDE, since elastic fibers are normal and increased in amount, while in dermal striae elastic fibers are curled and clumped at the edge of the lesion and reduced in the deep dermis enabling fat tissue hernia formation.^9^ Additionally, the two entities present contrasting clinical features. Thickening of the skin, both clinically and in HFUS, can be observed in other rare entities, such as acromegaly,^10^ or morphea.^11^ The former phenomenon, in addition to the obvious clinical diversity, is due to increased fibroblasts density and their activation in the reticular dermis. Therefore, the thicker skin in acromegaly differs from focal dermal elastosis in HFUS since the dermis is uniformly thickened without hypoechogenic areas. Wang et al.^10^ reported a different dermoscopic image compared to the one we observed in LOFDE. Subsequently, in the fibrotic phase of morphea, the so‐called “fibrotic beams” (white clouds), which correspond to sclerotic collagen bundles in the mid and deep dermis, can be seen in dermatoscopy.^11^, ^12^ HFUS on the other hand, shows hyperechogenic dermis, acoustic attenuation of the dermis, and the unclear boundary between the dermis and the subcutaneous fat.^11^

In conclusion, taking into account the clinical picture of LOFDE, it seems that both HFUS and dermoscopy may be helpful in the diagnosis of this rare entity, but in doubtful cases, histologic examination and histochemical staining will determine the cause of decreased hyperechogenicity of collagen fibers.

## CONFLICT OF INTEREST STATEMENT

No potential conflict of interest was reported by the authors.

## Data Availability

The data that supports the findings of this study are available in the supplementary material of this article (references).
